# A Floor-Map-Aided WiFi/Pseudo-Odometry Integration Algorithm for an Indoor Positioning System

**DOI:** 10.3390/s150407096

**Published:** 2015-03-24

**Authors:** Jian Wang, Andong Hu, Chunyan Liu, Xin Li

**Affiliations:** 1 School of Environmental Science and Spatial Informatics, China University of Mining and Technology, Xuzhou 221116, China; E-Mails: han_winter@cumt.edu.cn (A.H.); lcy_sia@163.com (C.L.); linuxcumt@126.com (X.L.);; 2 Sino-UK Geospatial Engineering Centre, The University of Nottingham, Nottingham NG7, 2RD, UK

**Keywords:** WiFi/pseudo-odometry, extended kalman filter, particle filter, floor map

## Abstract

This paper proposes a scheme for indoor positioning by fusing floor map, WiFi and smartphone sensor data to provide meter-level positioning without additional infrastructure. A topology-constrained K nearest neighbor (KNN) algorithm based on a floor map layout provides the coordinates required to integrate WiFi data with pseudo-odometry (P-O) measurements simulated using a pedestrian dead reckoning (PDR) approach. One method of further improving the positioning accuracy is to use a more effective multi-threshold step detection algorithm, as proposed by the authors. The “go and back” phenomenon caused by incorrect matching of the reference points (RPs) of a WiFi algorithm is eliminated using an adaptive fading-factor-based extended Kalman filter (EKF), taking WiFi positioning coordinates, P-O measurements and fused heading angles as observations. The “cross-wall” problem is solved based on the development of a floor-map-aided particle filter algorithm by weighting the particles, thereby also eliminating the gross-error effects originating from WiFi or P-O measurements. The performance observed in a field experiment performed on the fourth floor of the School of Environmental Science and Spatial Informatics (SESSI) building on the China University of Mining and Technology (CUMT) campus confirms that the proposed scheme can reliably achieve meter-level positioning.

## 1. Introduction

Indoor navigation has become an essential technique that can be applied in a number of settings, such as in a supermarket as a shopping guide, for a fire emergency service for navigation, or for a hospital patient for tracking. However, some techniques that have been successfully used that are similar to the Global Navigation Satellite System (GNSS) [1,2,3] are not suitable for indoor navigation. Real-time indoor positioning using existing techniques remains a challenge, and this is a bottleneck in the development of indoor location-based services (LBSs) [4].

The solution for indoor positioning is increasingly regarded as being based on the integration of multiple technologies, e.g., WiFi, ZigBee, inertial navigation systems (INSs), and laser scanning systems (LSSs). Each has its shortcomings, but an integrated system can combine the advantages of several of these technologies. Pahlavan and Li reviewed the technical aspects of the existing technologies for wireless indoor location systems [5]. There are two main hardware layouts that can be used in an indoor situation: (1) a sensor network, such as a WiFi or ZigBee system [6,7,8]; and (2) self-contained sensors, such as gyroscopes, accelerometers or magnetometers [9,10,11,12]. However, the stringent demands of reliable and continuous navigation in indoor environments are unlikely to be achievable using a single type of layout, and developing a hybrid scheme for reliable and continuous positioning is therefore a core prerequisite for real-time indoor navigation [13,14,15].

It is well recognized that trilateration and fingerprint matching are two basic WiFi-based approaches to locating an object in an indoor environment. In the first method, the user coordinates are calculated based on the distances between access points (APs) and the user. However, the distance measured based on the WiFi signal path loss model is so unstable that it is impossible to use such measurements in a practical indoor navigation system. Fingerprint matching is a more practical approach for use in a market-orientated indoor navigation system, and this technique has been widely researched, especially with the rapid market penetration of the modern smartphone. APs in supermarkets, schools, hospitals, and other infrastructures are also freely available for fingerprint database establishment. Artificial intelligence (AI) methods, e.g., decision trees and neural networks, constitute a new possible approach to determining a user’s location [16]. Nevertheless, some inevitable shortcomings exist, e.g., tedious fingerprint database updates and the need to alleviate the “go and back” phenomenon by integrating other techniques [5,16,17]. In addition, the cost of continuously using the WiFi radio on a mobile device can be prohibitive. Nonetheless, such methods are the focus of significant research efforts [12].

Pedestrian dead reckoning (PDR) algorithms, based on accelerometer, gyroscope and magnetometer measurements, can be used as a complementary method of developing an indoor navigation system. The basic PDR procedure involves step detection, step length estimation and heading determination [4,17]. In practice, acceleration measurements are an ideal choice for step detection, considering the periodicity of a pedestrian’s walking pattern, and there are three types of step detection algorithms: peak detection, flat-zone detection and zero-crossing detection. The deficiencies of the peak and zero-crossing detection algorithms create the potential for missing detection or over-detection if the thresholds are not appropriately set, and over-detection may also occur in the case of the flat-zone detection algorithm because the flat-zone test statistic varies with different walking patterns [18]. Considerable research has been conducted in an attempt to improve the accuracy of step length estimation, and the techniques that have been developed for this purpose can be summarized as constant/quasi-constant models, linear models, nonlinear models, and AI models [19]. A look-up table conveniently stores a few levels of step length for a given pedestrian based on his/her locomotion mode and the time duration of every step [20]. The linear relationship between step length and step frequency can be used to estimate step length. Kourogi and Kurata utilized the correlation between vertical acceleration and walking velocity to compute the walking speed and then estimated the step length by multiplying the walking speed by the time of the unit cycle of locomotion [17]. Cho presented a neural network for step length estimation that is unaffected by accelerometer bias and the acceleration of gravity [21]. A gyroscope and a magnetometer are two types of heading sensors that are typically used when the PDR algorithm is applied [22]. Klingbeil and Xiao proposed the concept of correcting the magnetic azimuth using gyro data collected over a short time, thereby allowing the heading angles to be estimated by combining gyroscope and magnetometer measurements [22,23]. A biaxial magnetic compass may be used to calculate the azimuth after compensating for the inclination of the compass using a shoe-mounted accelerometer [21]. The use of an INS/EKF framework to reduce heading drift has been demonstrated [11]. A detector has been proposed that can perform magnetic field measurements, which can be used for heading estimation with adequate accuracy. This detector utilizes different magnetic field test parameters that can be analyzed to produce good magnetic field measurements [24]. One factor that limits the use of PDR alone for indoor navigation is its susceptibility to cumulative errors over time. To improve the reliability and accuracy of a PDR navigation system, the gross error caused by the sensor’s raw observations must also be avoided. To this end, an electromyography (EMG) method was presented and compared with a traditional method based on accelerometers in several field tests, and the results demonstrated that the EMG-based method was effective and that its performance in combination with a PDR algorithm can be comparable to that of accelerometer-based methods [24,25].

To overcome these constraints, a floor map can be used to further calibrate the bias and correct for unreasonable positioning results. For example, combining gyroscope measurements with the use of a floor map allows the orientation to be corrected using only map aids [26,27], and large heading errors are eliminated via the long-range geometrical constraints exploited by particle filters (PFs) [28]. Extending these techniques to multiple floors and stairways could also be made possible by significantly adapting their constraints to suit pedestrians [29,30]. Unfortunately, the large number of particles makes it unrealistic to operate such algorithms in a real-time manner. However, the integration of several techniques can dramatically reduce the number of particles required in a PF model.

**Figure 1:**
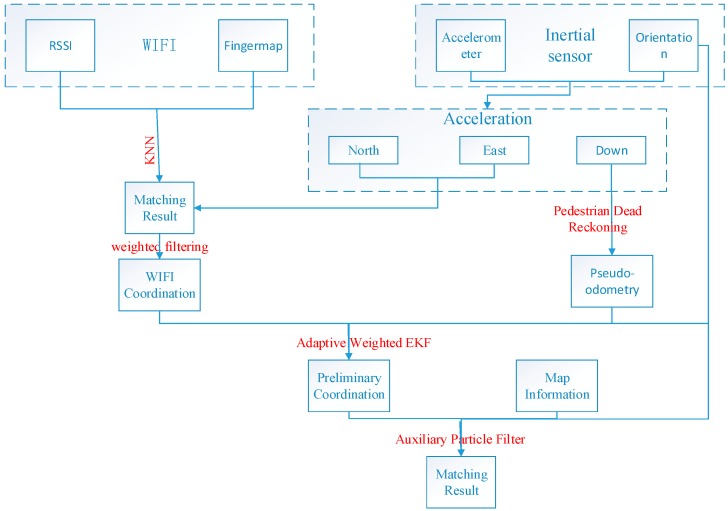
The general flow-chart.

To summarize, the methodologies of the whole article is concluded below:

(1) Theoretical analysis. A series of basic researches have been analyzed. As mentioned in the introduction, the signal-based network such as Fingerprint System, and INSs should be two fundamental techniques in indoor localization. However, the stringent demands of reliable and continuous navigation in indoor environments are unlikely to be achievable using a single type of layout, and developing a hybrid scheme for reliable and continuous positioning is therefore a core prerequisite for real-time indoor navigation. The aim is to overcome the drawbacks of conventional architectures at theoretical level to make it possible to improve the performance of an integrated WiFi/pseudo-odometry system.
(2) Integration Methodology Development. In the past, extended or unscented Kalman filter (EKF, UKF) and Particle Filter (PF) have mainly been used in data processing. However, in various cases as indicated in the introduction section, the situation is a little different. For instance, the noise dealt with by Kalman filter is assumed to be white noise, and PF algorithm requires a large amount of calculation. To overcome these constraints, a floor map can be used to further calibrate the bias and correct for unreasonable positioning results.
(3) Physical System Implementation and Tests. The specific course is shown in Figure 1 below:

In this paper, a scheme for indoor positioning by fusing floor map, WiFi and smartphone sensor data to obtain a real-time hybrid indoor navigation result is presented. Compared with the existing technology, Topology-Constrained KNN Positioning method introduced the floor map as a constraint, which could improve the accuracy and operational speed of the WiFi result. Besides, this method fits linear zones much better, such as corridors and narrow roads, which would be hard for GPS to fit, and the most useful places for WiFi localization technology. On the other hand, the multi-threshold PDR algorithm, presented in this paper, could clearly detect most steps accurately in the experiment. In addition, pseudo-odometry (P-O) is presented in this paper as a new exclusive term which means that by simulating the odometer, the step lengths are transformed to the time-domain (TD). The remainder of the paper is organized as follows: In Section 2, a topology-constrained KNN positioning algorithm is proposed, and Section 3 proposes a pseudo-odometry measurement simulation procedure based on a multi-threshold PDR algorithm. Subsequently, a WIFI/P-O integration scheme based on a fading-factor-based EKF is demonstrated in Section 4. Thereafter, Section 5 presents a scheme for floor-map-aided integration based on a PF, which is the core of the hybrid integration scheme. Finally, two experiments are analyzed in Section 6, and Section 7 concludes the paper.

## 2. Topology-Constrained KNN Positioning Algorithm

First, as the infrastructure of our indoor positioning approach, the signal fingerprint method, which is based on a WiFi technique, includes offline fingerprint database creation and online location matching. An area of interest is divided into regular lattices during offline database creation, and the corners of the lattice are used as the training samples for the reference points (RPs). The fingerprint database is also created by collecting the received signal strength indicator (RSSI) measurements of the available access Points (APs) and the corresponding coordinate values $\left(X_{RP},Y_{RP}\right)$ of the corners.

### 2.1. Topology-Constrained Fingerprint Database Creation

To improve the positioning accuracy, the geometric layout of an indoor floor map is modeled using a fingerprint database to a certain extent. For this task, the algorithm first segments the indoor floor map into sub-regions based on the specific building layout, and RP lattices of various shapes are clustered. Then, a topology-constrained fingerprint database is created by recording both the RSSI measurements and the geometric characteristics of the RPs. To be specific, the RSSI matrix $RP_{i}$ of the $i^{th}$ RP is given by

(1) $$RP_{i}=\left[\begin{array}{ccc} \begin{array}{c} \begin{array}{cc} P\left(A_{1}O_{1}\text{|}\left(\theta_{i}\text{|}C_{I}\right)\right) & P\left(A_{2}O_{1}\text{|}\left(\theta_{i}\text{|}C_{I}\right)\right) \end{array}\\ \begin{array}{cc} P\left(A_{1}O_{2}\text{|}\left(\theta_{i}\text{|}C_{I}\right)\right) & P\left(A_{2}O_{2}\text{|}\left(\theta_{i}\text{|}C_{I}\right)\right) \end{array} \end{array} & \begin{array}{c} \cdots \\ \cdots \end{array} & \begin{array}{c} P\left(A_{k}O_{1}\text{|}\left(\theta_{i}\text{|}C_{I}\right)\right)\\ P\left(A_{k}O_{2}\text{|}\left(\theta_{i}\text{|}C_{I}\right)\right) \end{array}\\ \begin{array}{cc} \vdots & \end{array}\vdots & \ddots & \vdots \\ \begin{array}{cc} P\left(A_{1}O_{v}\text{|}\left(\theta_{i}\text{|}C_{I}\right)\right) & P\left(A_{2}O_{v}\text{|}\left(\theta_{i}\text{|}C_{I}\right)\right) \end{array} & \cdots & P\left(A_{k}O_{v}\text{|}\left(\theta_{i}\text{|}C_{I}\right)\right) \end{array}\right]$$

where $A_{k}$ is the $k^{th}$ AP available in the sub-region. $\theta_{i}=\left(Pt_{i},GSS_{i}\right)$ denotes the coordinate and the topology relationship, namely, $Pt_{i}$ and $GSS_{i}$ represent the coordinate of the RP and the topology relationship, referred to as the Geometric Strength of the Sporadic Signal (GSSS), between the RP and the other adjacent RPs, respectively. *C_I_* is the *i^th^* (I=1,2,⋯N) cluster, where N is the total number of clusters. $O_{v}$ denotes the $v^{th}$ RSSI measurement with respect to sub-region $C_{I}$.

### 2.2. Topology-Constrained KNN Positioning Algorithm

It has been experimentally proven that the parameter K is not directly related to the positioning accuracy for the classical KNN fingerprint-database-based algorithm [31], and further research demonstrated that using a K parameter that has been corrected based on the indoor layout can improve the positioning accuracy [4]. In this paper, considering the RP topology in eight directions, a modified KNN algorithm, which chooses the value of K adaptively, is presented and implemented in a real-time indoor navigation system.

**Figure 2:**
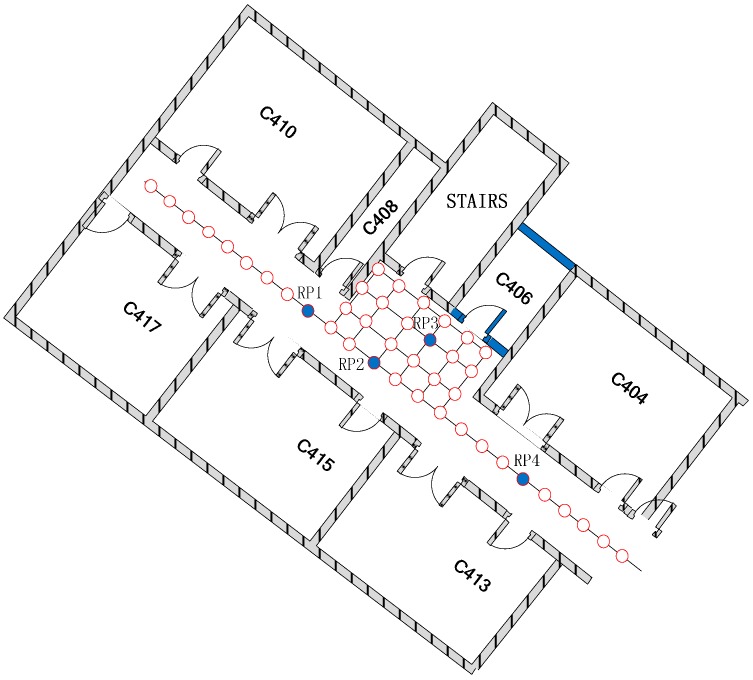
The equally spaced lattices for the Geometric Strength of the Sporadic Signal (GSSS).

The GSSS indicator of the $i^{th}$ reference point ($RP_{i}$), which is denoted by $GSS_{i}|O_{i}$ ($O_{i}=\left\{sn_{E},sn_{EN},sn_{N},sn_{NW},sn_{W},sn_{WS},sn_{S},sn_{SE}\right\}$), is used to describe the topology structure. First, a given element of $O_{i}$ should be set to null if no adjacent RP exists in the corresponding direction. Subsequently, $GSS_{i}$ is determined by summing the numbers of available RPs in all eight directions. For instance, $RP_{1}$, as marked in Figure 2, is adjacent to three RPs, and therefore, the corresponding GSSS value is 3; and those of $RP_{2}$, $RP_{3}$ and $RP_{4}$ are 5, 8 and 2, respectively.

**Figure 3:**
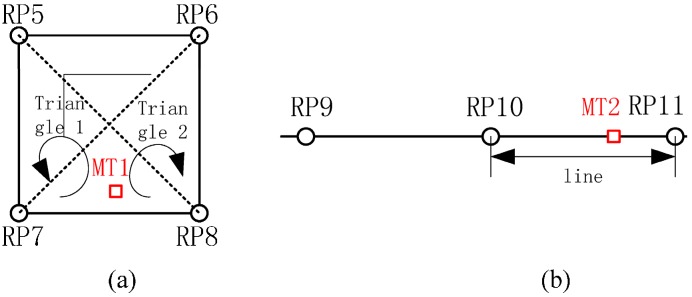
Optimal RP selection: (**a**) Optimal Triangle Selection; (**b**) Optimal Line Selection.

To further illustrate the modified KNN algorithm, suppose that MT1 in Figure 3a is a user coordinate, which must be estimated based on the surrounding RPs, $RP_{i}\left(i=5,\cdots ,8\right)$. Triangles RP5-RP7-RP8 and RP6-RP7-RP8 consist of RPs that can be simultaneously used to describe corresponding RP topologies. In this case, the *K* value of the KNN algorithm for the user MT1 is set to 3, and the estimated user coordinate is calculated as follows:

(2) $$\{\begin{array}{c} X_{MT}=\frac{\sum_{j=1}^{K}\left(r_{j}\times \left(X_{RP_{j}}\text{|}C_{I}\right)\right)}{\sum_{j=1}^{K}r_{j}}\\ Y_{MT}=\frac{\sum_{j=1}^{K}\left(r_{j}\times \left(Y_{RP_{j}}\text{|}C_{I}\right)\right)}{\sum_{j=1}^{K}r_{j}} \end{array}$$

where $\left(X_{MT},Y_{MT}\right)$ denotes the estimated user coordinate and $\left(X_{RP_{j}},Y_{RP_{j}}\right)$ is the $j^{th}$ RP coordinate of the K RPs. $r_{j}$ is the correlation coefficient between the RSSI matrix of the $j^{th}$ RP in the fingerprint database and the user’s RSSI matrix measured in real time. In addition, K may also be set to 4 if the distance between MT1 and the center of the square is less than a given threshold. In the scenario depicted in Figure 3b, RP10 and RP11 are used to describe the topology, and the position of MT2 is calculated using the modified KNN algorithm with *K* = 2.

For a given RP, the GSSS value varies from 1 to 8 in differently shaped lattices. Considering the calculation load, K should be set to 2 when GSS≤2 but to 3 or 4 when GSS>2. Overall, the procedure for indoor positioning using the modified KNN algorithm is summarized in Figure 3. Of the elements illustrated in this chart, the offline topology-constrained fingerprint database should be regarded as the highest priority. During online location determination, the user is required to record the RSSI measurement, which is used to determine the corresponding sub-region. Thereafter, the topology calculation is performed using the method described above. The K value and the corresponding RPs are also essential for the topology-constrained KNN algorithm to be able to produce the desired positioning results.

**Figure 4:**
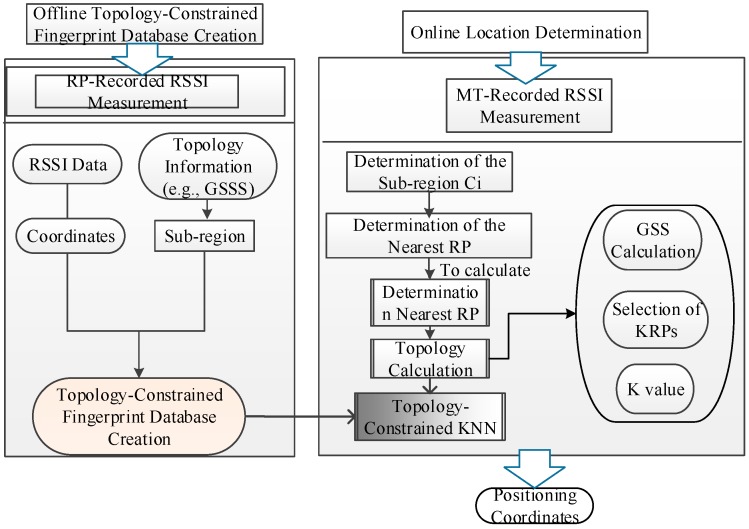
Topology-constrained K nearest neighbor (KNN) positioning algorithm.

The flow chart of the topology-constrained KNN positioning algorithm is summarized in Figure 4. The offline topology-constrained fingerprint database is created and includes RSSI data and the corresponding coordinates, topology information for each RP and pre-set sub-region information depending on the floor map layout. In the online coordinate calculation phase, user-recorded RSSI measurements are first used to match the corresponding sub-region and determine the nearest RP. Thereafter, GSSS and K are calculated with respect to a specific RP. The RPs are eventually determined and used as input to the KNN algorithm to calculate the user positions.

## 3. Simulation of P-O Measurements

Despite the importance of WiFi data, there is a second indispensible input to this method, namely, inertial data (from inertial sensors), which will be introduced below in detail. It is widely known that pedestrian dead reckoning (PDR) algorithms, which are based on the number of footsteps and the step length, have recently begun to be implemented more widely. Moreover, a heading should be obtained as a value in the 2D plane and should be estimated based on measurements collected by a gyro and magnetometer, whereas the floor level can be detected in advance through barometer measurements. Section 3.1 and Section 3.2 illustrate a new multi-threshold step detection algorithm and a hybrid heading estimation algorithm. Section 3.3 presents the flow chart of the P-O measurement simulation.

### 3.1. Multi-Threshold Step Detection

The maximum time duration of a step and the minimum and maximum changes in the acceleration magnitude during one step are frequently used as parameters in techniques for avoiding faulty step detection. The dynamic time warping (DTW) algorithm provides further improvement in step detection accuracy [32]. In this paper, a multi-threshold algorithm is proposed to detect steps based on raw acceleration measurements. The amplitude *a* of the extremum of an acceleration signal can be used to determine the stance or walking status of an individual. The step detection is terminated if the individual is stationary, and otherwise, a set of parameters of the multi-threshold algorithm is used for step detection. If $Num_{pk}$ and $Num_{vy}$ denoted the numbers of detected peaks and valleys, then a multi-threshold algorithm for peak detection can be described as follows:

If only one peak is detected, *i.e.*, $Num_{pk}-Num_{vy}=1$,

(3) $$Step_{p}=\{\begin{array}{l} 1,\delta_{t_{pv}}=\frac{1}{2}\delta_{t_{p}},a_{p}\geq \delta_{a_{p}}＆\Delta t_{p}\geq \delta_{\Delta t_{p}}＆\Delta t_{pv}\geq \delta_{\Delta t_{pv}}\\ 0,\delta_{\Delta t_{pv}}=\frac{1}{2}\delta_{\Delta t_{p}},a_{p}<\delta_{a_{p}}||\Delta t_{p}<\delta_{\Delta t_{p}}||\Delta t_{pv}<\delta_{\Delta t_{pv}} \end{array}$$

**Figure 5:**
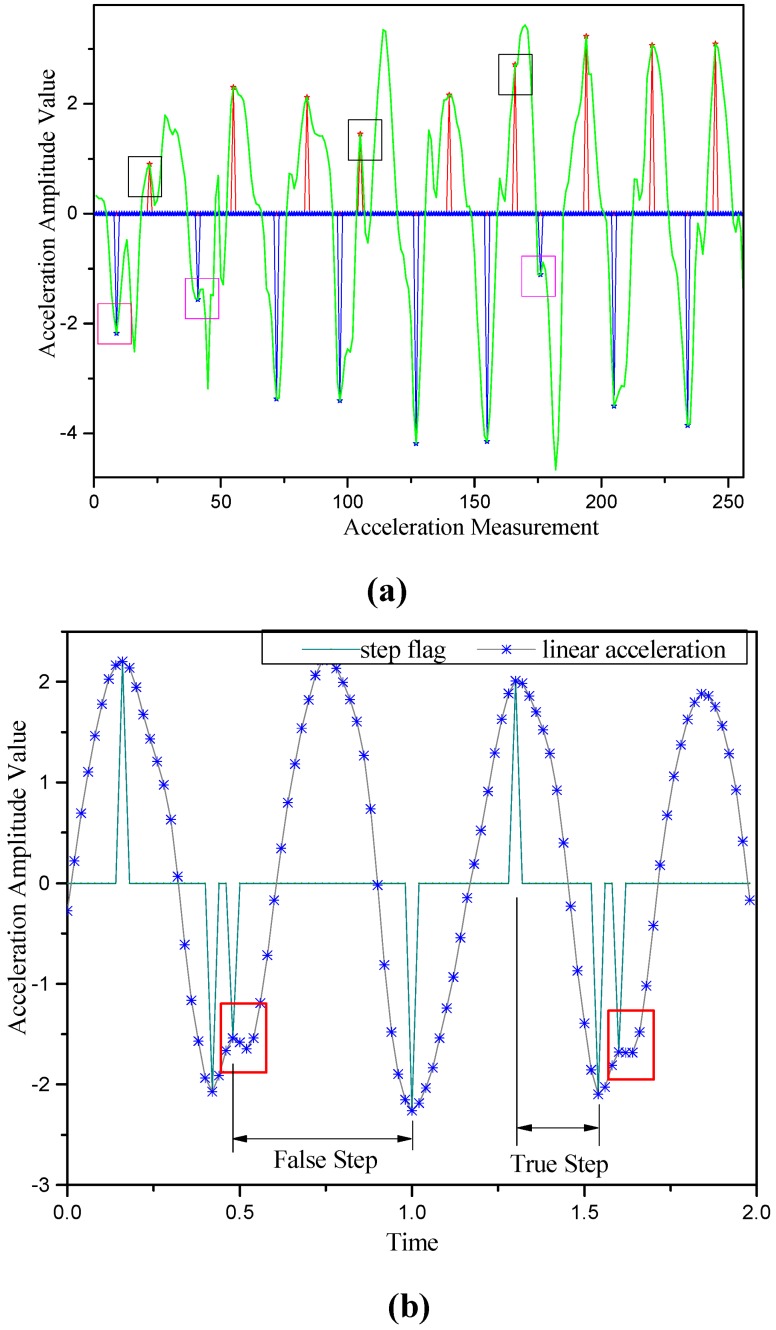
Faulty step detection induced by a pseudo-peak and a pseudo-valley: (**a**) Pseudo-peak and pseudo-valley detection; (**b**) Pseudo-step detection.

If two consecutive peaks are detected, *i.e.*, $Num_{pk}-Num_{vy}=2$, then the true peak can be detected as follows:

(4) $$Step_{p}=\{\begin{array}{l} 1,\delta_{\Delta t_{pv}}=\frac{1}{2}\delta_{\Delta t_{p}},a_{p}\geq \delta_{a_{p}}＆\Delta t_{p}\geq \delta_{\Delta t_{p}}＆\Delta t_{pv}\geq \delta_{\Delta t_{pv}}＆\Delta a_{p}\geq 0\\ 0,\delta_{\Delta t_{pv}}=\frac{1}{2}\delta_{\Delta t_{p}},a_{p}<\delta_{a_{p}}||\Delta t_{p}<\delta_{\Delta t_{p}}||\Delta t_{pv}<\delta_{\Delta t_{pv}}||\Delta a_{p}<0 \end{array}$$

where $a_{p}$ is the extremum of the peaks in the acceleration signal. $\Delta a_{p}$ is the difference between the consecutive peak values. $\Delta t_{p}$ is the time difference between the consecutive peaks. $\Delta t_{pv}$ is the time difference between a consecutive peak and valley. $\delta_{a_{p}},\delta_{\Delta t_{p}}$ and $\delta_{\Delta t_{pv}}$ are the threshold values for peak detection, which are determined empirically. For example, $\delta_{\Delta t_{p}}$ is set to 0.2 s as an empirical value. If $Step_{p}$ is equal to 1, then a true peak is detected; otherwise, it is a false peak. as Figure 5a.

The parameters $\left(a_{v},\Delta a_{v},\Delta t_{v},\Delta t_{vp}\right)$ can also be used to construct a multi-threshold algorithm for valley detection. If $Num_{pk}-Num_{vy}=0$,

(5) $$valley=\{\begin{array}{c} 1,\delta_{\Delta t_{vp}}=\frac{1}{2}\delta_{\Delta t_{v}},a_{v}\geq \delta_{a_{v}}＆\Delta t_{v}\geq \delta_{\Delta t_{v}}＆\Delta t_{vp}\geq \delta_{\Delta t_{vp}}\\ 0,\delta_{vp}=\frac{1}{2}\delta_{\Delta t_{v}},a_{v}<\delta_{a_{v}}||\Delta t_{v}<\delta_{\Delta t_{v}}||\Delta t_{vp}<\delta_{\Delta t_{vp}} \end{array}$$

Similar to the case of peak detection, if two consecutive valleys are detected, *i.e.*, $Num_{vy}-Num_{pk}=1$, then the true valley can be detected as follows:

(6) $$valley=\{\begin{array}{c} 1,\delta_{\Delta t_{vp}}=\frac{1}{2}\delta_{\Delta t_{v}},a_{v}\geq \delta_{a_{v}}＆\Delta t_{v}\geq \delta_{\Delta t_{v}}＆\Delta t_{vp}\geq \delta_{\Delta t_{vp}}＆\Delta a_{v}\geq 0\\ 0,\delta_{\Delta t_{vp}}=\frac{1}{2}\delta_{\Delta t_{v}},a_{v}<\delta_{a_{v}}||\Delta t_{v}<\delta_{\Delta t_{v}}||\Delta t_{vp}<\delta_{\Delta t_{vp}}||\Delta a_{v}<0 \end{array}$$

where $a_{v}$ is the amplitude of the valley extremum. $\Delta a_{v}$ is the difference between the two consecutive $a_{v}$ values. $\Delta t_{v}$ is the time difference between the consecutive valleys. $\Delta t_{vp}$ is the time difference between a consecutive valley and peak. $\delta_{a_{v}},\delta_{\Delta t_{v}}$ and $\delta_{\Delta t_{vp}}$ are the thresholds for valley detection, where $\left|\delta_{a_{v}}\right|=\left|\delta_{a_{p}}\right|$ and $\delta_{\Delta t_{v}}=\delta_{\Delta t_{p}}$. If *valley* is equal to 1, then a true valley is detected; otherwise, it is a false valley, as Figure 5b.

**Figure 6:**
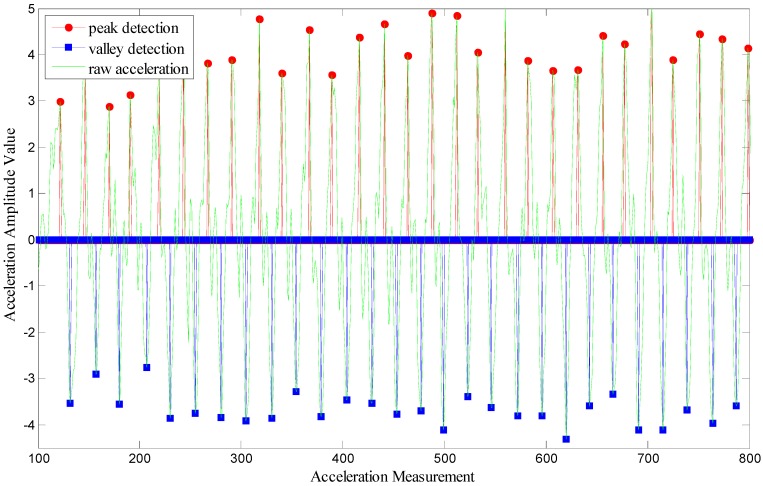
Step detection using raw acceleration data.

With the method we display above, we can find out the result of step detection as Figure 6.

Step length estimation is an important factor that affects the positioning accuracy and can be performed in combination with a step detection procedure. The step length is related to the acceleration and is typically given by

(7) $$L_{k}=K\times \sqrt[{4}]{acc_{max}-acc_{min}}$$

where $L_{k}$ is the length of the $k^{th}$ step. $acc_{max}$ and $acc_{min}$ are the minimum and maximum amplitudes, respectively, of the acceleration. The value of the coefficient K depends on the individual and can be calibrated.

### 3.2. Hybrid Heading Estimation

Heading determination is a significant component of PDR-based positioning. The heading angle ψ is defined as the angle of rotation about the z axis with respect to the horizon/ground, which can be estimated using a gyroscope integrated with a magnetometer. The improved heading estimation algorithm presented by Wonho Kang is applied here [33]. The fused heading angle is calculated as follows:

(8) $$\left\{ \begin{array}{l} \theta_{k}=\alpha \theta_{k-1}+\beta \theta_{m,k}+\gamma \theta_{g,k},\;\;\;\;\theta_{\Delta ,c}\leq \theta_{c},\theta_{\Delta ,m}\leq \theta_{m}\\ \theta_{k}=\beta \theta_{m,k}+\gamma \theta_{g,k},\;\;\;\;\;\;\;\;\;\;\;\;\;\;\;\;\;\;\;\;\;\theta_{\Delta ,c}\leq \theta_{c},\theta_{\Delta ,m}>\theta_{m}\\ \theta_{k}=\alpha \theta_{k-1},\;\theta_{\Delta ,c}>\theta_{c},\theta_{\Delta ,m}\leq \theta_{m}\\ \theta_{k}=\alpha \theta_{k-1}+\gamma \theta_{g,k},\;\theta_{\Delta ,c}>\theta_{c},\theta_{\Delta ,m}>\theta_{m} \end{array}\right.\;\;\theta_{\Delta ,c}=\left|\theta_{m,k}-\theta_{g,k}\right|,\theta_{\Delta ,m}=|\theta_{m,k}-\theta_{m,k-1}|$$

where *m* and *n* denote the magnetometer and the gyroscope. *α*, *β* and *γ* are the weights of the current measurements from the gyroscope and the magnetometer. $\theta_{m,k}$ and $\theta_{g,k}$ denote the measurements acquired by the gyroscope and the magnetometer, respectively, for the $k^{th}$ step. $\theta_{m}$ is the standard deviation of the magnetometer, and $\theta_{c}$ is the correlation between the magnetometer and the gyroscope. $\theta_{\Delta ,c}$ is the difference between $\theta_{m,k}$ and $\theta_{g,k}$. $\theta_{\Delta ,m}$ is the difference in the magnetometer reading between two consecutive steps *k* and *k*−1.

**Figure 7:**
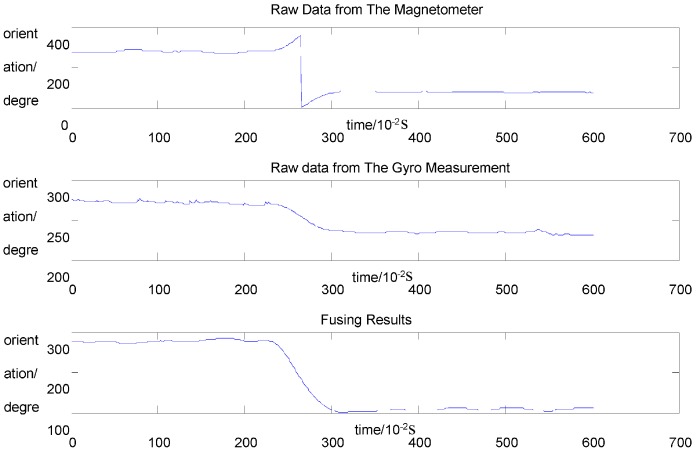
A test of heading fusion.

Figure 7 shows the fusion results of a test in which a person walked forward five steps with a smartphone held firmly on his hand, stopped for a brief period, and eventually turned around and returned on the same path. The results reveal that the pseudo-heading measurements recorded by the magnetometer before and after the turn are considerably smoothed by the proposed algorithm.

### 3.3. P-O Measurement Simulation

**Figure 8:**
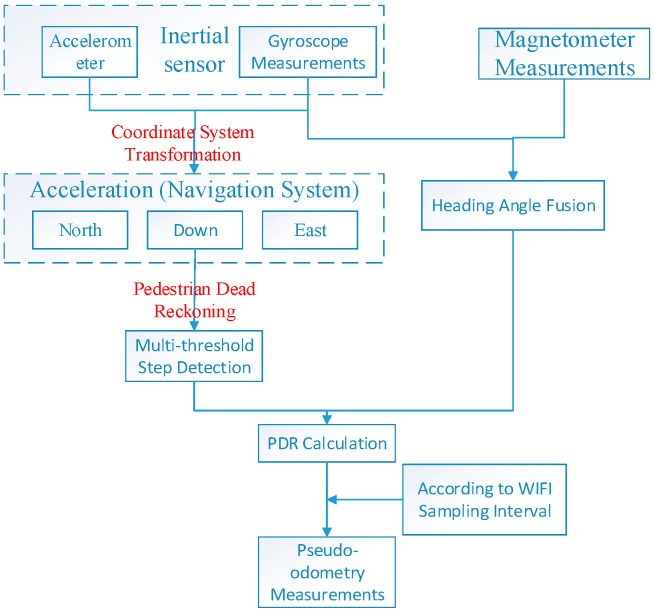
Flow chart of P-O measurement simulation.

Pseudo-odometry (P-O) is introduced to determine how far a mobile user terminal departs from a designated starting point, which can yield a relative distance measurement for indoor positioning. P-O measurements can be easily simulated by sampling the PDR distance in accordance with the rate of RSSI measurements, which are considered to be collected once every 2 s in this paper. Figure 8 presents a flowchart that summarizes the procedure for P-O measurement simulation.

## 4. WiFi/P-O Integration Based on a Fading-Factor-Based EKF

Despite the beneficial features of the approaches described above, the absolute coordinates obtained using the WiFi algorithm may exhibit a “go and back” phenomenon, which is tedious for user applications. P-O measurements can yield a relatively fluid user trajectory but suffer from accumulated positioning errors. A reliable integration algorithm must be adopted to alleviate the disadvantages of both the WiFi and P-O algorithms. A fading-factor-based EKF can accommodate the significant differences between the WiFi and P-O approaches and appropriately balance their weights; such an approach is proven to be effective in this paper.

### 4.1. Dynamic Equation

The system state is estimated using recursive EKF equations, and the state equation is [34]

(9) X(k+1)=F(X(k))+ΓW(k)

Its discretization is

(10) X(k+1)=ϕ(k)X(k)+Γ(k)W(k)

where

(11) $$\phi \left(k\right)=\left[\begin{array}{cc} \begin{array}{cc} 1 & 0\\ 0 & 1 \end{array} & \begin{array}{cc} t & 0\\ 0 & t \end{array}\\ \begin{array}{cc} 0 & 0\\ 0 & 0 \end{array} & \begin{array}{cc} 1 & 0\\ 0 & 1 \end{array} \end{array}\right]$$

(12) $$\text{Γ}={\left[\begin{array}{cc} \frac{t^{2}}{2} & \begin{array}{ccc} 0 & t & 0 \end{array}\\ 0 & \begin{array}{ccc} \frac{t^{2}}{2} & 0 & t \end{array} \end{array}\right]}^{t}$$

$\text{X}\left(k\right)=\left[\begin{array}{ccc} N\left(k\right) & E\left(k\right) & \begin{array}{cc} v_{e}\left(k\right) & v_{n}\left(k\right) \end{array} \end{array}\right]$; N(k) and E(k) are the position states in the northern direction and the eastern direction, respectively, at time *k*; $v_{e}\left(k\right)$ and $v_{n}\left(k\right)$ are the velocity states in the eastern and northern directions, respectively, at time *k*; and W(k) is the system noise at time *k*. F(X(k)) and Γ are the system transition functions. ϕ(k) and Γ(k) are the discretizations of F(X(k)) and Γ, respectively. *t* is the time interval.

### 4.2. Observation Equation

The observation equation for the integrated positioning based on WiFi, pseudo-odometry and magnetometer measurements can be written as follows:

(13) Z(k)=h(X(k))+V

Its discretization can be written as

(14) Z(k+1)=H(k)X(k)+V

where $\text{Z}\left(k\right)=\left[\begin{array}{ccc} N_{wifi} & E_{wifi} & \begin{array}{ccc} s_{e} & s_{n} & heading \end{array} \end{array}\right]$; $N_{wifi}$ and $E_{wifi}$ are the position information obtained via WiFi in the northern and eastern directions, respectively; and $s_{e}$ and $s_{n}$ are the P-O increments of two consecutive samples in the eastern and northern directions, respectively; heading are the heading angle. h(X(k)) is the *k*^th^ recursive filter observation function, and *V* is the measurement noise vector.

### 4.3. Fading-Factor-Based EKF

The estimates of the state vector from the extended Kalman filter can be obtained by performing a time update and a measurement update at a given instant of time:

(15) $${\overset{˰}{X}}_{k}={\overset{˰}{X}}_{k,k-1}+G_{k}\left(Z_{k}-h\left(X\left(k\right)\right)\right)$$

(16) $$G_{k}=P_{k,k-1}H_{k}^{T}{\left(H_{k}P_{k,k-1}H_{k}^{T}+V_{k}R_{k}V_{k}^{T}\right)}^{-1}$$

(17) $${\overset{˰}{X}}_{k,k-1}={\text{Φ}}_{k,k-1}{\overset{˰}{X}}_{k-1}$$

(18) $$P_{k,k-1}={\text{Φ}}_{k}P_{k-1}{\text{Φ}}_{k}^{T}+Q_{k}$$

(19) $$P_{k}=\left(I-G_{k}H_{k}\right)P_{k,k-1}$$

where $G_{k}$ is the gain matrix of the extended Kalman filter at time *k*, $H_{k}$ is the *k*^th^ recursive filter observation matrix, $P_{k}$ is the covariance matrix of the state vector at time k, $R_{k}$ is the covariance matrix of the measurement noise vector at time k, $Q_{k}$ is the covariance matrix of the system noise at time k, and the subscript *k*, *k*−1 represents the state or covariance estimate from time *k*−1 to time k.

Through these five equations above, the ${\overset{˰}{X}}_{k}$ and $P_{k}$, which represents the status variable and covariance of the equations, respectively, could keep innovating as time goes by. Eventually, we could come to accurate coordination and velocity to a certain extent. However, as time advances, the old data lose their value generally through the improvement of accuracy. Therefore, a fading factor is required to define the weight among the observed value.

To balance the dynamic equation and the observation equation, a fading-factor-based EKF is used to overcome the deficiencies of the classic EKF to obtain more reliable navigation results [35]. Furthermore, the fading factor should be a self-adapting number to allow each variable condition to be managed accurately. The algorithm is written below:

(20) $$\lambda_{k}=max\left\{1,\frac{1}{n}tr\left(N_{k}M_{k}^{-1}\right)\right\}$$

(21) $$M_{k}=H\left(k+1\right)\phi_{k,k-1}\sum^{\text{​}}{\overset{˰}{X}}_{k-1}\phi_{k,k-1}^{T}H{\left(k+1\right)}^{T}$$

(22) $$N_{k}=\sum^{\text{​}}{\bar{V}}_{k}-H\left(k+1\right)\sum^{\text{​}}W_{k}H{\left(k+1\right)}^{T}-R$$

where $\lambda_{k}$ represents the optimal solution for this formula, namely, the fading factor. $\sum^{\text{​}}{\bar{V}}_{k}$ represents the variance of the predicted residuals. $\sum^{\text{​}}{\overset{˰}{X}}_{k-1}$ is the variance of the optimal solution, and R denotes the measurement noise matrix. The modified covariance matrix modified is updated as follows:

(23) $$\{\begin{array}{c} P_{k,k-1}=\lambda_{k}{\text{Φ}}_{k}P_{k-1}{\text{Φ}}_{k}^{T}+Q_{k}\;\;\;\;\;\;\left|S_{WIFI}-S_{PDR}\right|<\delta S\\ P_{k,k-1}={\text{Φ}}_{k}P_{k-1}{\text{Φ}}_{k}^{T}+Q_{k}\;\;\;\;\;\;\;\;\;\;\;\left|S_{WIFI}-S_{PDR}\right|>\delta S \end{array}$$

where $P_{k}$ is the covariance matrix of the state vector at time *k* and $Q_{k}$ is the covariance matrix of the system noise at time *k*. $S_{WIFI}$ and $S_{PDR}$ are the increments of the WiFi and PDR coordinates, respectively. δS is a threshold that is used to define whether the WiFi coordinate has “go and back”, and if $\left|S_{WIFI}-S_{PDR}\right|<\delta S$, then the WiFi coordinate will be considered to be reliable and will be input into the fading-factor-based EKF, where $\lambda_{k}$ is given by Formula (20).

### 4.4. WiFi/P-O Integration Based on the Fading-Factor-based EKF

**Figure 9:**
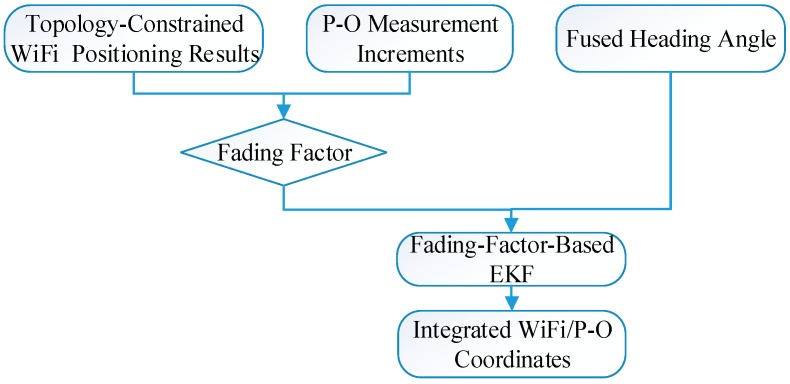
WiFi/P-O integration using the fading-factor-based extended Kalman filter (EKF).

The flow chart for WiFi/P-O integration based on the fading-factor-based EKF is summarized in Figure 9. The topology-constrained WiFi positioning results and the P-O measurement increments are used to determine the fading factor, and the heading angle obtained by fusing the gyroscope and magnetometer measurements is simultaneously input into the observation equation. Then, the final positioning result is obtained using the fading-factor-based EKF model.

**Figure 10:**
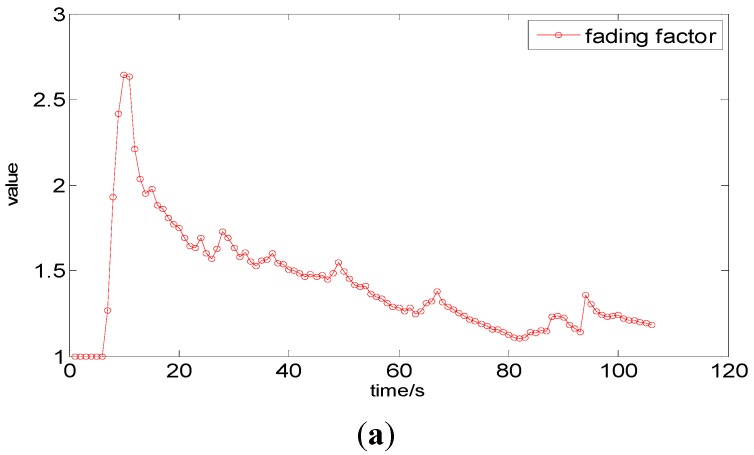

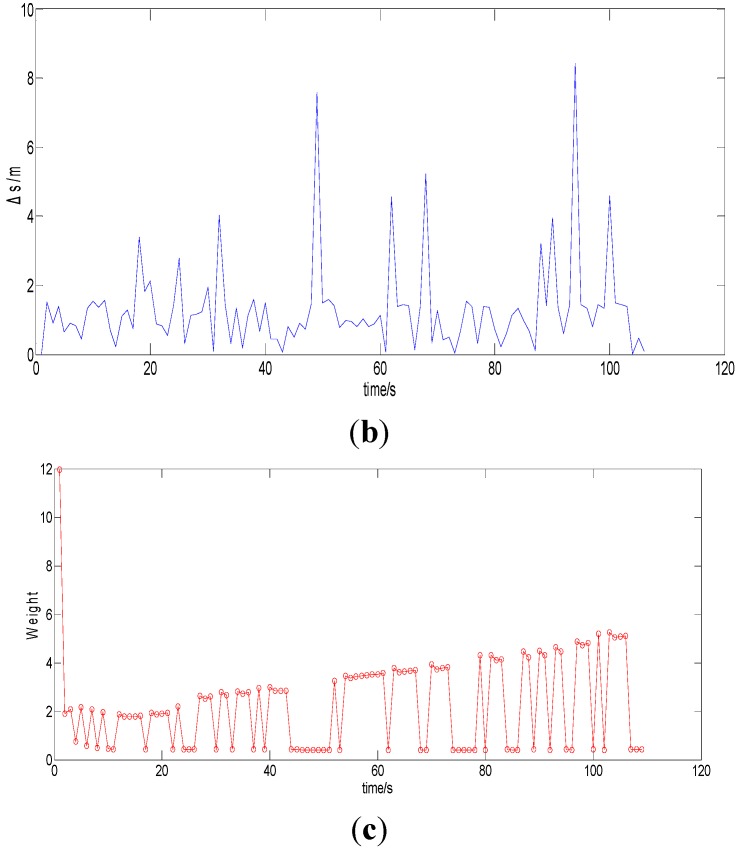
Performance of the fading-factor-based EKF: (**a**) The variation in the fading factor; (**b**) The variation in δS; (**c**) The variation in the prediction weight value.

The variation in the fading factor variation with respect to time in an experiment that is described in Section 6 is shown in Figure 10a, and the corresponding variation in the threshold δS is depicted in Figure 10b. The variation in the prediction weight of the position vector is shown in Figure 10c. It is illustrated in Figure 10 that the fading factor ultimately converges, and an initial sharp growth of the fading factor from 1 to a peak value of greater than 2.5 is caused by large errors in the WiFi positioning coordinates. Thereafter, it continues to decrease down to a value of approximately 1. Compensation for the dramatic changes in δS is provided by the weight value, which is adaptively adjusted through Equation (23). The value of 3 m selected for δS in this paper can eliminate almost all the gross error in the WiFi or P-O measurements.

## 5. Floor-Map-Aided Integration Based on a PF

Because the error may not be Gaussian in many situations, particle filters have recently seen widespread use in indoor positioning. However, such an approach typically requires nearly hundreds of times the number of calculations required by other filters because of the difficulty of the particle selection. Particle filters cannot be applied for real-time or near-real-time operation using a smartphone or other miniaturized devices. In this paper, a floor map is used to alleviate the computational load by constraining the particle numbers and providing more reliable location data.

### 5.1. Particle Filter

The posterior probability density Function (PDF) of a state x(k) given an observation Z(k) can be approximately written as follows, according to Branko (2004) [36]:

(24) $$\text{p}\left(x\left(k\right)\text{|}Z\left(k\right)\right)\approx \overset{N}{\underset{i=1}{\sum }}w^{i}\left(k\right)\delta \left(x\left(k\right)-x^{i}\left(k\right)\right)$$

where p(·) and δ(·) represent the posterior probability and the Dirac function, respectively. N is the total particle number corresponding to a specific filter step *k*. $x^{i}\left(k\right)$ is the $i^{th}$ particle of state x(k), and $w^{i}\left(k\right)$ is the corresponding weight value. The sum of the *N* weights, $\overset{N}{\underset{i=1}{\sum }}w^{i}\left(k\right)$, is equal to 1.

A particle filter has the merit of being applicable to a non-linear and non-Gaussian noise model. Multiple particle filters are available with varying performance; the classic auxiliary sampling importance resampling (ASIR) algorithm is used in this paper. This filter comprises the following six steps [37]:

(1) Initialization: The particle filter begins by generating N particles $\left({\text{x}}^{i}\left(0\right),\text{i}=1\cdots \text{N}\right)$ based on the initial PDF p(x(0)).
(2) Prediction: a new ${\text{x}}^{i}\left(k+1\right)$ is obtained from the transition PDF $\text{p}\left(x^{i}\left(k+1\right)\text{|}x^{i}\left(k\right)\right)$.
(3) Importance Sampling: For any particle $x^{i}\left(k+1\right)$, the weight is $w^{i}\left(k+1\right)=p\left(z\left(k+1\right)\text{|}x^{i}\left(k+1\right)\right)$ which actually defined by the distance between its prediction position and observed value.
(4) Normalization: The weight values of the $k^{th}$ steps are normalized as follows: $w_{k}^{i}=\frac{w_{k}^{i}}{\sum_{i=1}^{N}w_{k}^{i}}$, to set the weight to a normative standard, which could be useful for the resampling and updating.
(5) Resampling and Particle Updating: The normalized weights are used to resample the particles. Particles with high weights are duplicated, and particles with low weights are deleted. $\overset{n}{\underset{i=1}{\sum }}w_{k+1}^{i}\geq u$, where u represents a random number between 0 and 1 and *n* represents the number of particles to be resampled in this step. $x_{k+1}^{j}=x_{k+1}^{n}\left(j=1,\cdots ,N\right)$, and the updated particles are given by $x_{k+1}^{j}=mean\left(x_{k+1}^{j}\right)$.
(6) Calculation of Results: The classic ASIR algorithm is used to obtain the filter results.

The accuracy and stability of the particle filter primarily depend on the number of particles used, but as the number of particles increases, the amount of calculation and the calculation time increase. For the given particle filter, the mean square error (MSE) and the calculation time (CT) are shown for various particle numbers (PNs) in Table 1.

**Table 1:** The mean square error (MSE) and calculation time (CT) for various particle numbers (PNs) for the particle filter.

PN	100	200	300	400	500
MSE (m^2^)	4.34	3.58	3.25	3.09	3.12
CT (s)	1.218	2.867	6.536	10.798	16.048

**Figure 11:**
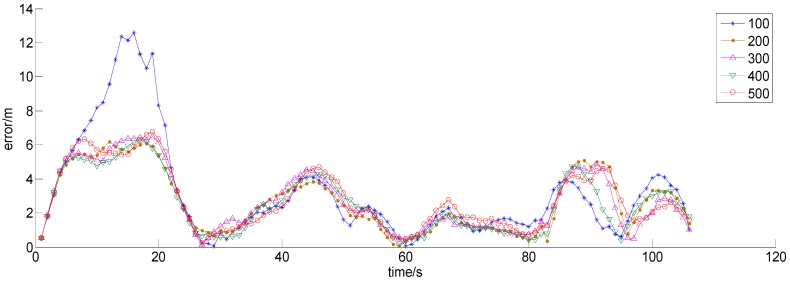
The error maps for various particle numbers.

Figure 11 is also useful in the attempt to determine an appropriate particle number for a simulation. Once the particle number reaches 300, the MSE is reduced to approximately 3 m, and the calculation time increases to 6.536 s. Afterward, the MSE remains nearly unchanged with an increasing number of particles, although the calculation time continues to increase.

### 5.2. Map-Aided Particle Filter

A floor map records the positions of the walls and the obstacles in an indoor scenario, which may restrict the movements of the particles and provide more reliable position information. If the predicted position of a particle passes outside the effective region of the map, then the corresponding weight of the particle filter is set to zero:

(25) $${\text{w}}_{k}^{i}=\{\begin{array}{l} 0\;\;\;\;\;\;\;\;\;\mathrm{if}\;\mathrm{the}\;\mathrm{particle}\;\mathrm{id}\;\mathrm{outside}\;\mathrm{the}\;\mathrm{effective}\;\mathrm{region}\\ \frac{1}{\sqrt{2\pi }\sigma }e^{\left[-\frac{{\left(x^{i}\left(k\right)-x\left(k\right)\right)}^{2}+{\left(y^{i}\left(k\right)-y\left(k\right)\right)}^{2}}{2\sigma^{2}}\right]}\;\;\;\;\;\;\;\;\;\;\;\;\;\mathrm{otherwise} \end{array}$$

where x(k) and y(k) denote the N and E coordinates, respectively. *σ* represents the variance of the results of the EKF algorithm, which is set to 1 m in this paper. A test similar to that presented in Section 5.1 demonstrates that the floor-map-aided PF algorithm can provide more accurate and stable results in a shorter amount of time and using a considerably smaller number of particles; a calculation time of less than one second can be achieved using mobile phones (Table 2).

**Table 2:** The MSE and CT for various PNs for the map-aided particle filter.

PN	40	50	60	70	80
MSE (m^2^)	2.890	2.599	2.601	2.615	2.640
CT (s)	0.492	0.564	0.692	0.793	0.966

### 5.3. Scheme for Floor-Map-Aided Integration Based on a PF

**Figure 12:**
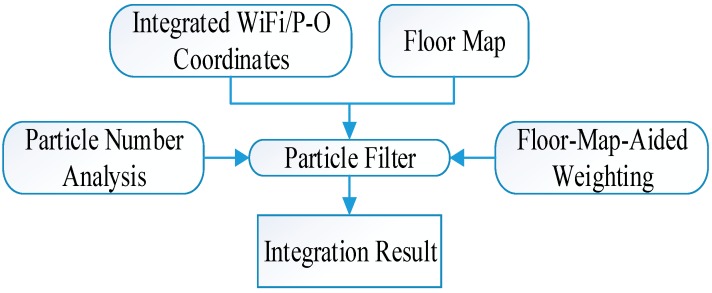
Scheme for floor-map-aided integration based on a PF.

The flow chart for floor-map-aided integration using a particle filter is summarized in Figure 12. The number of particles must be decided before the particle filter is used to integrate the integrated WiFi/P-O coordinates and the floor map, and the floor-map-aided weighting formula given by (20) is also used to avoid the “go and back” phenomenon. The detailed performance of the proposed scheme will be demonstrated in Section 6.

## 6. Experimental and Analysis Section

### 6.1. TEST One

**Figure 13:**
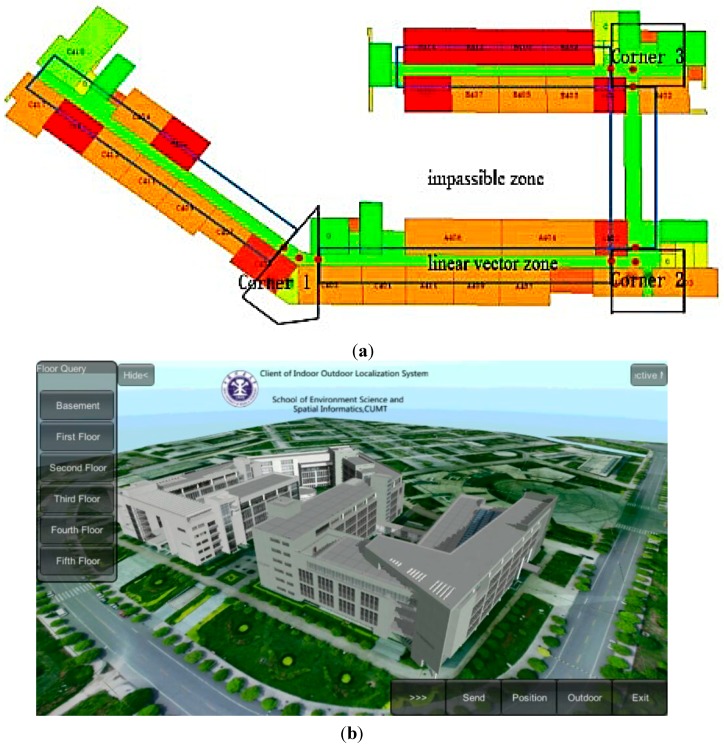
Experimental site: (**a**) floor map of the fourth floor; (**b**) 3D model of the experimental site.

**Figure 14:**
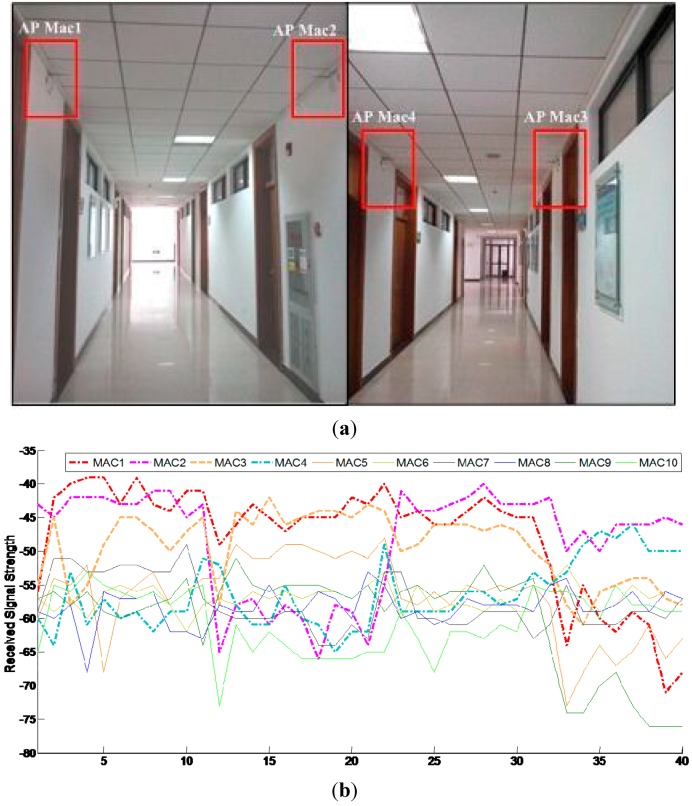
APs located in the corridor: (**a**) 60 APs located along the corridor; (**b**) RSSI data of 10 available APs for an RP.

To verify the effectiveness of the proposed algorithm, a field experiment was performed on the fourth floor of the School of Environmental Science and Spatial Informatics (SESSI) building on the campus of China University of Mining and Technology (CUMT) in Xuzhou, Jiangsu, China. In Figure 13, blue outlines indicate linear corridors, black outlines indicate non-linear corridors, and other areas are unavailable for a person to pass through. Sixty APs in total are distributed along the corridors on the ceiling (Figure 14). A SAMSUNG GALAXY S4 was used as the user terminal in the experiment; its technical specifications are shown in Table 3.

**Table 3:** Technical specifications of the SAMSUNG GALAXY S4 smartphone.

Instrument	Scale Factor	Random Walk
Accelerometer (m/s^2^)	19.6133	±0.0006
Gyroscope (m/s^2^)	8.7266	±0.0003
Magnetometer (μT)	1200.0000	±0.0600

To compare the indoor positioning results, four schemes were designed:

Scheme 1: Topology-constrained KNN positioning test (GCK).
Scheme 2: PDR-based indoor positioning test (PDR).
Scheme 3: Integrated WiFi/P-O indoor positioning test using fading-factor-based EKF (WPO).
Scheme 4: Floor-map-aided integrated WiFi/P-O positioning test (MWPO).

The EKF was used to fuse the information from different sensors for Schemes 1–3. In Scheme 4, the PF was used for floor-map-aided modeling, and the number of particles was set to 100. The smartphone was held as stable as possible during the experiment and thus was considered to be synchronized with the human body’s motion. The floor level should, in general, be determined in advance from barometer measurements, although only two-dimensional positioning was considered here.

**Figure 15:**
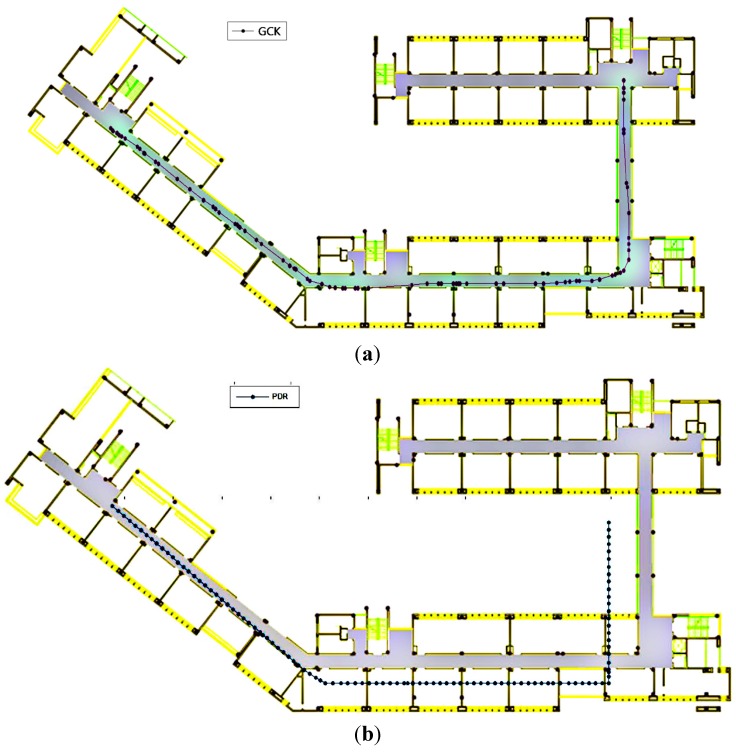

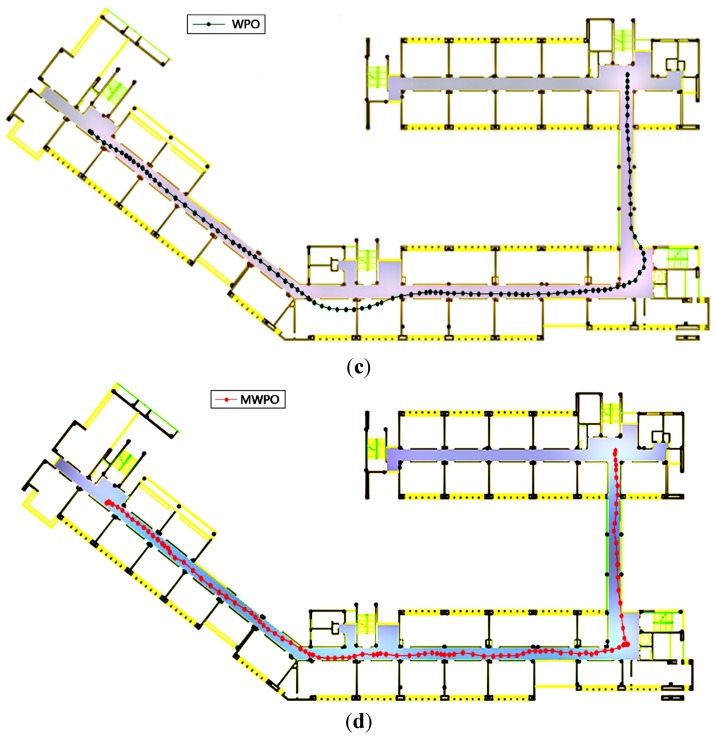
Estimated position: (**a**) WiFi with fingerprint database; (**b**) PDR; (**c**) WiFi/P-O integration; (**d**) Map-aided WiFi/P-O integration.

Figure 15 shows the position trajectories for the four schemes. The colorful dots represent the paths of travel calculated using the different schemes. In the test, the test participant walked with a uniform and stable gait; therefore, the true trajectory should be stable.

Figure 15a demonstrates that the positioning accuracy achieved using WiFi is often related to the intensity of the points from the fingerprint database. The accumulated errors in the locations and the “go and back” phenomenon adversely affect the positioning results.

**Figure 16:**
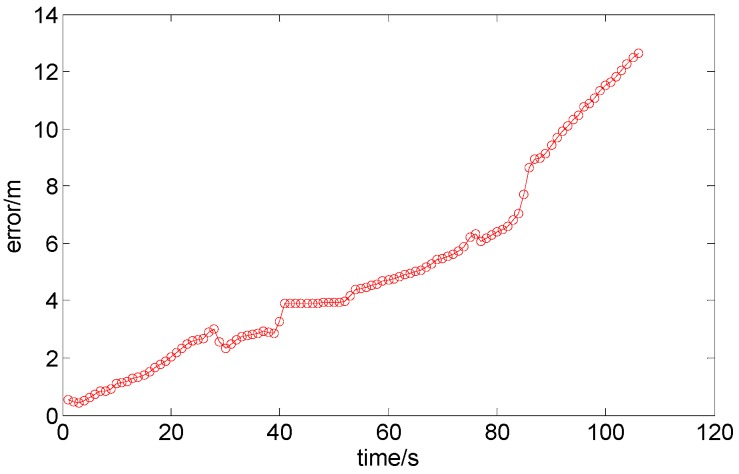
The accumulated error of the PDR algorithm.

The PDR algorithm, the results of which are shown in Figure 15b, inevitably incurs cumulative errors, as shown in Figure 16. Over time, the accumulated error increases, although this error could be mitigated by the floor-map-aided algorithm.

As seen from Figure 15c, the positioning results of the WPO algorithm are more evenly distributed and rarely suffer from the “go and back” phenomenon. The results illustrate that the EKF fusion algorithm preserves the continuity and stability of the PDR algorithm while simultaneously restricting the accumulation of error, thereby improving the positioning accuracy. Moreover, some positioning results shown in Figure 15c that are located in impassable areas are excluded when the floor-map-aided integrated WiFi/P-O positioning algorithm is used, as shown in Figure 15d.

**Figure 17:**
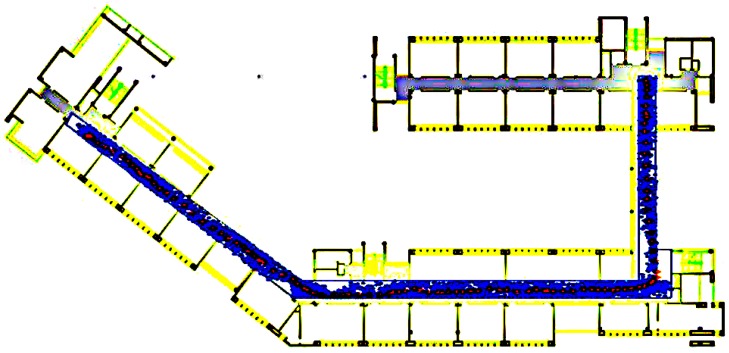
The particle distribution of the MWPO algorithm.

As observed in Figure 17, the particle distribution of the MWPO algorithm is restricted to lie within the accessible region. In this case, the particle sample number was set to 50. For single-point positioning, the calculation time of this algorithm is notably shortened, as analyzed in Section 5.2, enabling it to run on a smartphone.

**Figure 18:**
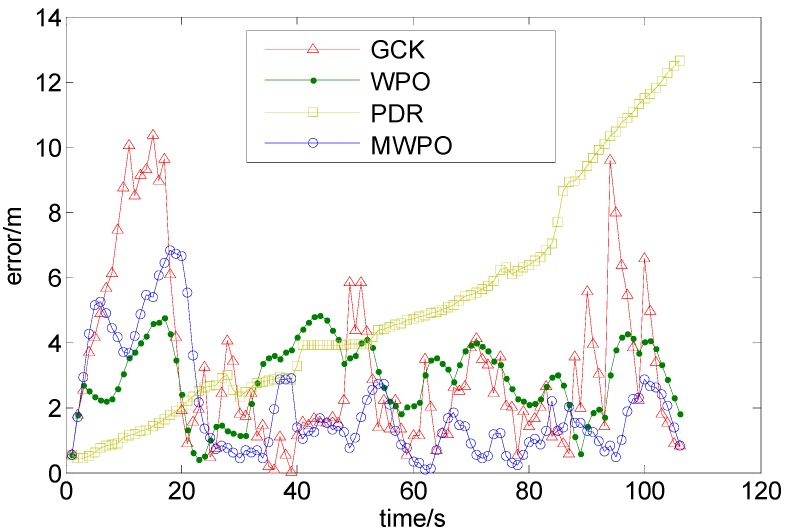
The error time series of the four methods.

Figure 18 shows the time series of the position errors of the four schemes with respect to the reference positions provided by a master station. The largest errors of the MWPO integration algorithm were observed during the first 20 steps, which may have been caused by the large WiFi positioning errors.

The statistics of the MSE, the average error (AE) and the maximum error (ME) reveal that the map-aided algorithm achieved the most reliable and accurate positioning results. In this test, the accuracy indicated by the MSE of the integrated WPO indoor positioning algorithm with EKF was improved by 22.9% compared with the pure WiFi algorithm, and the AE and ME were reduced by 7.4% and 53.5%, respectively. Compared with the PDR algorithm, the MSE of the integrated WPO algorithm was improved by 48.1%, and the AE and ME were reduced by 41.8% and 61.8%, respectively. The integrated MWPO positioning algorithm was more accurate than the WPO algorithm, with an MSE improvement of 4.9%, an AE reduction of 18.0% and a similar ME (Table 4).

**Table 4:** Error analysis of the four schemes.

ERROR	GCK	PDR	WPO	MWPO
MSE/m	4.037	5.993	2.618	2.491
AE/m	3.135	4.990	2.344	1.922
ME/m	10.375	12.650	4.708	4.664

As seen from the comparison, the integrated MWPO positioning algorithm results in an improvement in the accuracy, reliability, and calculation rate and a decrease in the accumulated error; therefore, this method is superior to the others to a certain extent.

### 6.2. Test Two

To demonstrate the robustness of the MWPO integration algorithm for a smartphone-based indoor positioning system, gross errors were added to the WiFi observations as listed in Table 5.

**Table 5:** Gross errors added to the WiFi measurements.

Time	40 s	60 s	80 s
X direction (m)	10	10	0
Y direction (m)	0	0	10

**Figure 19:**
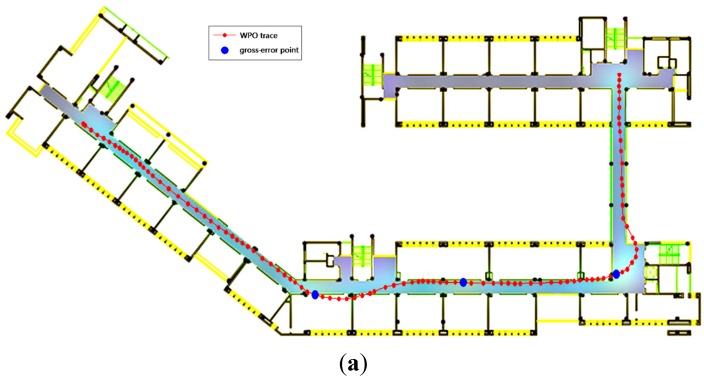

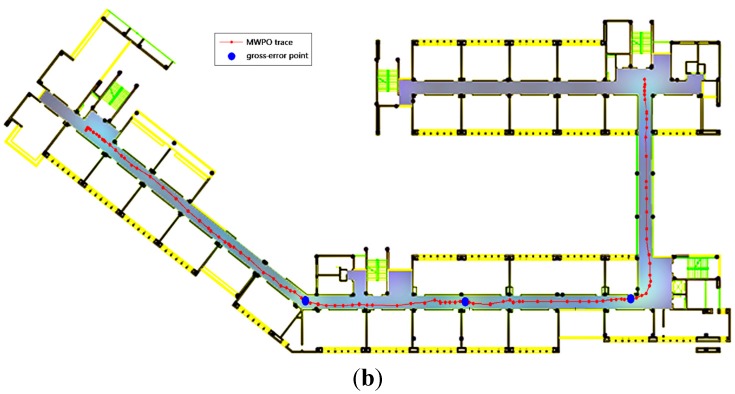
Estimated position: (**a**) WiFi/PDR integration with EKF; (**b**) Map-aided WiFi/P-O integration with PF.

Figure 19 illustrates the positioning trajectories determined using the WPO algorithm and the MWPO algorithm. As shown in Figure 19a, the three gross-error-contaminated points cause the trajectory to deteriorate around the affected epochs as a result of EKF recursion. In Figure 19b, the gross-error effects are entirely mitigated with the introduction of the MWPO algorithm. Table 6 shows the residuals of these two algorithms for each affected time point (40 s, 60 s and 80 s), and the results demonstrate that the MWPO algorithm achieves much higher reliability and accuracy.

**Table 6:** Residuals of the gross-error epochs.

Time	WPO	MWPO
40 s	4.8224 m	1.8483 m
60 s	3.5404 m	1.4832 m
80 s	3.0557 m	0.6947 m

**Figure 20:**
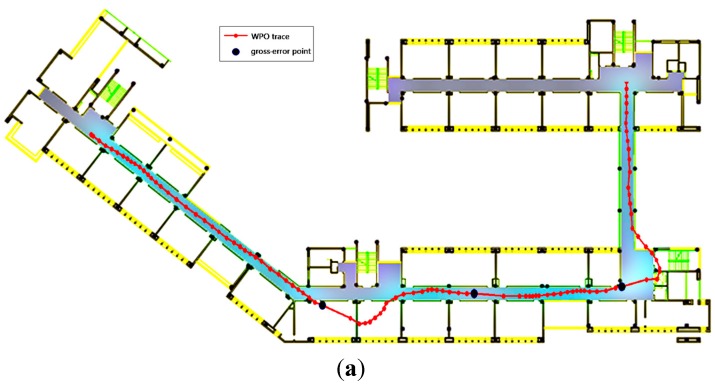

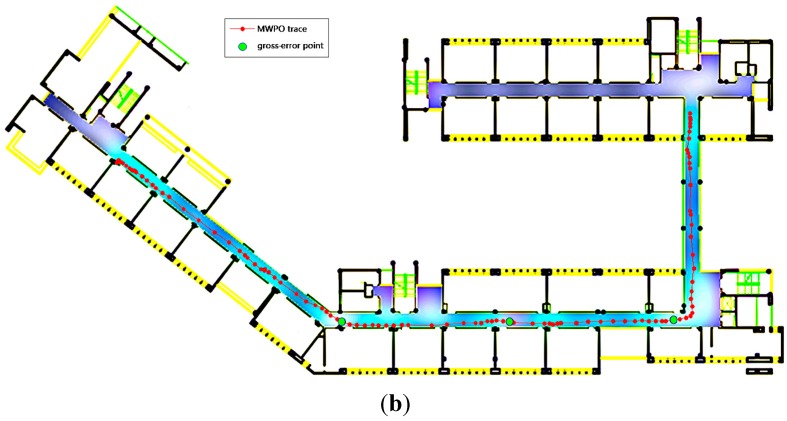
Estimated position: (**a**) WiFi/PDF integration with EKF; (**b**) Map-aided WIFI/P-O integration with PF.

Gross errors were also added to the P-O measurements as listed in Table 7 to verify the robustness of the WPO and MWPO algorithms. The resulting positioning trajectories obtained using the WPO algorithm and the MWPO algorithm are shown in Figure 20. As shown in Figure 20a, the three gross-error-contaminated points cause the trajectory to deteriorate around the affected epochs as a result of EKF recursion, similar to Figure 20a, and again, the gross-error effects are mitigated in the case of the MWPO algorithm. Table 8 lists the residuals of the gross-error-contaminated epochs.

**Table 7:** Gross errors added to the P-O measurements.

Time	40 s	60 s	80 s
s (m)	10	10	10

**Table 8:** Residuals of the gross-error epochs.

Time	WPO	MWPO
40 s	9.6667 m	1.1344 m
60 s	8.5872 m	2.0504 m
80 s	6.3026 m	1.3445 m

These results demonstrate that the MWPO algorithm is more robust than the WPO algorithm, regardless of where the gross error originates.

These observations yield the following conclusions regarding the investigated positioning algorithms for indoor navigation systems.

(1) A topology-constrained K nearest neighbor (KNN) algorithm and a multi-threshold PDR algorithm are treated as the fundamental basis of this methodology, and P-O measurements are simultaneously simulated. This approach represents an innovation in the attempt to combine WiFi and PDR data by providing a common axis (time axis) other than the step duration.
(2) The position determined based on WiFi data experiences fluctuations to some extent, particularly in certain locations, such as windows, glassed-in rooms and corridors. It was experimentally demonstrated that with the addition of supplementary data from inertial sensors, this fluctuation can be considerably decreased, thereby improving the reliability of the algorithm.
(3) Considering the issue of the fanning out of positions at corners, a fading factor was incorporated to improve the rate of convergence, thereby decreasing the fluctuation to an acceptable level and improving the accuracy of the entire algorithm.
(4) Despite the high accuracy provided by particle filters, a particle filter always requires a considerable amount of calculation, and therefore, most experiments using PFs require a central computer to cope with the information obtained from the nodes. However, incorporating an electronic map of the structure in question into the analysis eliminates redundant operations, thereby helping to improve computational speed and quality.
(5) With the development of methods of integrating the data from both WiFi and inertial sensors (P-O measurements), decreasing fluctuations (including rebound) and increasing particle filtering efficiency (map aid), the proposed hybrid algorithm succeeds in combining the measured data within a common time axis, improving the positioning reliability and producing a dramatic upward shift in operating rate and quality. Therefore, it offers the possibility of quasi-real-time measurements using mobile phones instead of a central computer, as has generally been used in other recent experiments.

## 7. Conclusions

This paper investigated several positioning algorithms for indoor navigation systems. A topology-constrained K nearest neighbor (KNN) algorithm and a multi-threshold PDR algorithm were presented, and P-O measurements were simulated. The proposed floor-map-aided WiFi/P-O integration algorithm combines the complementary advantages of all three techniques using a particle filter (PF) model. The experimental test results indicate that the integration of these techniques can not only avoid the “cross-wall” phenomenon but also the gross-error effects inherent to WiFi and P-O measurements. It was demonstrated that a higher indoor positioning precision can be achieved using a smaller number of particles when a floor map is used. The further development of the algorithm will focus on improving the calculation efficiency to allow it to run more efficiently on a smartphone system.
